# Selective Leptin Insensitivity and Alterations in Female-Reproductive Patterns Linked to Hyperleptinemia during Infancy

**DOI:** 10.1371/journal.pone.0059937

**Published:** 2013-03-27

**Authors:** Mariana Schroeder, Noga Kronfeld-Schor, Aron Weller

**Affiliations:** 1 Psychology Department, Bar Ilan University, Ramat Gan, Israel; 2 Gonda (Goldschmied) Brain Research Center, Bar Ilan University, Ramat Gan, Israel; 3 Zoology Department, Tel Aviv University, Tel Aviv, Israel; CUNY, United States of America

## Abstract

The dramatic increase in the prevalence of childhood obesity worldwide makes the investigation of its early developmental stages and effective prevention strategies an urgent issue. CCK_1_ deficient OLETF rats are a model of obesity previously used to study the early phases of this disorder. Here, we exposed wild type (LETO) females to an early obesogenic environment and genetically obese OLETF females to a lean postnatal environment, to assess long term alterations in leptin sensitivity, predisposition to diet induced obesity and adult female health. We found that genetically lean females reared by obese mothers presented early postnatal hyperleptemia, selectively reduced response to leptin and sensitivity to diet induced obesity when exposed to a high palatable diet as adults. The estrous cycle structure and intake profile were permanently disrupted, despite presenting normal adiposity/body weight/food intake. Genetically obese females reared by lean dams showed normalized early levels of leptin and reduced body weight, food intake and body fat at adulthood; normalized estrous cycle structure and food intake across the cycle, improved hormonal profile and peripheral leptin sensitivity and a remarkable progress in self-control when exposed to a high fat/palatable diet. Altogether, it appears that the early postnatal environment plays a critical role in determining later life coping with metabolic challenges and has an additive effect on the genetic predisposition that makes OLETF females morbidly obese as adults. This work also links, for the first time, alterations in the leptin system during early development to later life abnormalities related to female reproduction and health.

## Introduction

Maternal obesity during pregnancy and lactation may predispose the offspring to increased adiposity, accompanied by higher leptin and glucose levels later in life, especially in the presence of a genetic tendency [1], [2]. The maternal nutritional and hormonal environment during the perinatal period thus programs life-long appetite and metabolism in the offspring. The hormonal, neuronal and autocrine mechanisms mediating the maintenance of energy balance develop during this period, making it of high relevance in determining the programming of later sensitivity or resistance to obesity [3].

Leptin is mainly secreted by adipocytes in direct proportion with the amounts of adipose tissue in the body [4]. Its provides the central nervous system with information about adipose stores to enable the brain to make the adjustments necessary to balance energy intake and expenditure [5]. Early hyperleptinemia (caused by either maternal undernutrition or exogenous administration) can potentially induce peripheral leptin resistance in adult offspring [6], [7], [8]. Leptin signaling impairment, frequently found in obesity [9], [10], [11], has been associated with sensitivity to diet induced obesity, especially when animals are exposed to high fat/highly palatable diets [12]. In addition, leptin has a permissive role in initiating puberty and is crucial in the pathogenesis of reproductive dysfunction in several disease states of energy imbalance, such as anorexia on one side, and obesity on the other. Leptin interacts with the reproductive axis at multiple sites, stimulating at the hypothalamus and pituitary and inhibiting at the gonads. In conditions with excess energy stores or metabolic disturbances, elevated serum or follicular fluid leptin levels have been reported, raising the possibility that leptin deficiency or resistance may be at least partly responsible for the reproductive abnormalities usually found in these conditions [13].

The Otsuka Long Evans Tokushima fatty (OLETF) rat is a model of early onset hyperphagia induced obesity [14], [15]. As a result of the lack of CCK_1_ receptors [16] the short term satiety signal produced by CCK when fat is consumed [17] is ineffective. Previous studies showed that OLETF pups presented high adiposity during lactation, accompanied by a spontaneous leptin surge around postnatal (PND) 7 [15]. The relevance of this model relies in the fact that its obesity results from hyperphagia and can be completely prevented by food restriction [18], [19] in contrast to other genetic models [20], [21], [22]. Basal hypothalamic mRNA levels of the long form of leptin receptor (Ob-Rb) (the one that mediates intracellular signaling) at adulthood, do not differ between OLETF and LETO (Long Evans Tokushima Otsuka) control males [23] even though OLETF males appear to develop peripheral leptin resistance around the age of 8 weeks [24]. While the leptin system has never been examined in OLETF females, the high circulating levels of leptin appear to affect the estradiol/progesterone-induced luteinizing hormone (LH) and prolactin (PRL) surges in the proestrus phase of the estrous cycle, leading to fertility problems. This can be partially improved by food restriction leading to normalized levels of leptin [25]. Finally, the cycle of OLETF females is abnormal in structure, and intake across the cycle is not sufficiently decreased towards the estrous phase [26].

We performed two different studies examining the contribution of the early postnatal environment to obesity in the offspring [27], [28] using the cross-fostering strategy. We found that OLETF dams made pups obese through high fat milk and increased predisposition to nurse [27], while LETO dams reared lean pups [28]. Long term influences included some moderation in parameters of the metabolic syndrome in OLETF and hyperadiposity, hyperphagia and dyslipidemia in LETO females reared by obese dams, implying deep long lasting effects of the perinatal environment on their health [28].

On the basis of those results, the present study explored a potential mechanism underlying long term effects of early environment on adult phenotype. We exposed LETO pups to an obesogenic postnatal environment and OLETF pups to lean dams. This lead to four experimental groups: LETO controls, OLETF controls, OLETF dams rearing LETO pups (OdLp) and LETO dams rearing OLETF pups (LdOp). We aimed to, first, examine the potential of the early environment in determining lifelong sensitivity to diet induced obesity resulting from alterations in leptin sensitivity; and second, to assess the effects of early overnutrition and hyperleptinemia on reproductive health parameters in female offspring, as adults.

## Materials and Methods

### Ethics statement

The research protocol was approved by the Institutional Animal Care and Use Committee of Bar-Ilan University (Permit Numbers: 10-03-07, 08-03-10), and it adhered to the guidelines of the Society for Neuroscience.

### Subjects

OLETF and LETO rats were raised in the SPF facility of the Gonda Brain Research Center at Bar-Ilan University, Israel. LETO rats were chosen as controls, since the OLETF strain is an outbred line originating in that specific (Long Evans) colony and are considered as normal. Offspring were housed with their dams and litters until weaning and in pairs from then and on, in polycarbonate cages (23.5 cm height×26.5 cm width×43 cm length), with stainless steel wire lids, wood shavings as bedding and plastic tubes for enrichment. Food (2018S Teklad Global, 5% fat) and water were freely available. The animals were on a 12∶12 hr light∶dark cycle (lights on at 06:00) and 22+/−2°C room temperature. Newborn litters found until noon each day were designated as PND0. On PND1, litters were culled to 10 pups (minimum 8), with sex distribution kept as equal as possible. At this point, 2–3 females were fostered to a dam from the opposite strain and mixed with the rest of the litter. The research protocol was approved by the Institutional Animal Care and Use Committee of Bar-Ilan University (Permit Numbers: 10-03-07, 08-03-10), and it adhered to the guidelines of the Society for Neuroscience. All tests were performed on the diestrous phase of the estrous cycle.

### Experimental procedure

#### Body weight and intake

Rats were weighed every fifth day from birth until PND90. Intake was assessed daily from pairs of same-condition females starting at the time of weaning (PND22).

#### Nursing test

Nursing was determined as described [29]. Nursing of pups was examined once with their foster dam in their home cages, between 18–20 days postpartum. Experiments took place between 10:00 and 15:00 hrs, after a 4 hr separation from the dam. Pups were placed as a group in a cage containing bedding from the home cage and in a humid and warm incubator (33°C) for that period while the dams remained in their home cage. Excretion was induced with a cotton swab and then pups were weighed and individually identified with a permanent marker. After the isolation period, the pups were returned to the nest, scattered in the 4 corners of the cage (e.g., as in [29]) and weight gain (% of BW) and nursing time were recorded.

#### Leptin sensitivity test

Test (modified from [24], [30]) took place around PND60 (females always in the diestrous phase of their cycle). At 14 hr, food was removed from the cages for a two-hour deprivation period. At 16 hr, rats were injected with either murine leptin (i.p.) at a concentration of 2 mg/kg (Peprotech) or saline. All females underwent both treatments in the diestrous phase of their cycles in a random order. Intake was assessed 4 and 24 hours after injection. Results were calculated by deducting the females' intake after leptin injection by the amounts consumed after injection of saline.

#### High fat ENSURE challenge

Around PND75, rats received a choice of standard chow (5% fat), ENSURE vanilla (20% fat) and ENSURE plus vanilla (30%) overnight for a period of 12 hours without food deprivation. Ensure (Abbot) is a liquid rich in calories, very attractive to rats and frequently used in studies. The test was conducted for 4 days, with 2–3 days break for estrous changes. Food preference during the first and the following trials, as well as total kcal intake were examined.

#### Physiological measures

Rats were sacrificed on PND7, PND22 (weaning) or on PND90. On PND7 blood was collected after decapitation for determination of plasma leptin levels. At weaning, pups were anesthetized (pentobarbital (200 mg/ml) and Assival (5 mg/ml) (1∶1)), weighed, measured (body length and waist circumference) and scanned using Dual energy X-ray absorptiometry (DEXA; Lunar Piximus II). Percentage body fat, lean body mass, bone mineral density (BMD), bone mineral content (BMC) and bone area were assessed. On PND90 animals were decapitated after the scan, and trunk blood for leptin and insulin analysis was collected in chilled heparinized vacutainer tubes coated with EDTA. Brains were extracted and immediately frozen on dry ice. Plasma and brains were stored at −80°C.

#### Leptin, insulin, TNFα, IL 1β and IL 6

Plasma hormones were assessed using commercial ELISA kits. Leptin (R&D Systems, intra-assay variance 2.70, inter-assay variance 5.76, minimum detectable amount [MDA] 22 pg/ml), insulin (Linco, intra-assay variance 1.91, inter-assay variance 7.63, MDA 0.2 ng/ml), TNFα, IL 1β and IL 6 (Peprotech, intra-assay variance 2.31–4.22, inter-assay variance 4.68–6.93, MDA 0.2–0.4 ng/ml) ELISAs were determined according to the manufacturers' instructions.

#### Steroid metabolites extraction from feces

The protocol was modified from previous protocols [31], [32], [33]. Briefly, fresh feces were collected between 12–14PM, two-four hours after vaginal smears sampling. Feces were stored in absolute ethanol and preserved at −20°C until analysis. The day of the assay, samples were dried in a hood overnight, triturated and weighed. Ten ml of ethanol 96% were added to each sample (0.2–0.5 gr), followed by incubation overnight at room temperature in glass tubes. The next day, samples were centrifuged for 10 minutes at 2000×g; supernatants were collected with a Pasteur pipette and transferred to new glass tubes. Feces were then dried in a speed vac, reconstituted with the kits running buffer and diluted for steroid analysis. The extract of each fecal sample was diluted 1∶16, according to results from pre-tested samples. Estradiol and progesterone metabolites were assessed using commercial ELISA kits (Cat.No.582601 for progesterone and Cat.No.52251 for Estradiol, Cayman). Quality-control samples were run during the assays, and the intra assay precision of our samples was in the range of the kit standards. The final concentration obtained was divided by the weight of the original sample for normalization. Estradiol intra-assay variance was 12.3, inter-assay variance was 5.5, and MDA was 20 pg/ml. Progesterone intra-assay variance was 4.9, inter-assay variance was 1.5, and MDA was 10 pg/ml.

#### Estrous cycle

The estrous cycle of 50–80 day old females was examined daily in the morning as previously described [26]. Samples were collected by introduction and immediate extraction of a small amount of phosphate buffer with a micropipette in the rat's vagina. The stage of the estrous cycle (diestrous 1 or metaestrous, diestrous 2, pro-estrous or estrous) was determined by examining the appearance and abundance of cells within the vaginal cytology samples.

#### Immunohistochemistry

In a separate set of animals (N = 5–6) (aged 60–70 days), food was removed at 14 hr for a two-hour deprivation period. At 16 hr, rats were injected with either murine leptin (i.p.) at a concentration of 2 mg/kg (Peprotech) or saline and were perfused 90–100 minutes after the leptin/saline injection. Rats were anesthetized with pentobarbital sodium (200 mg/ml) and Assival (5 mg/ml) (1∶1), and transcardially perfused with heparinized saline containing 2% sodium nitrite, followed by a fixative containing 4% paraformaldehyde in 0.1 M phosphate buffer. Brains were removed and post-fixed in perfusing fixative for 24 hs and in 35% sucrose solution for 2 weeks at 4°C. The hypothalamic slices containing the ARC, DMH and NTS were cut (35 µm) using a freezing cryostat (−20°C) (ARC: Bregma −3.30 to −2.12, DMH: −3.30 to −2.56 and NTS: −14.08 to −13.68 respectively) according to Paxinos & Watson, 1997). Free-floating brain sections were stained for c-fos immuno-reactivity using the avidin-biotin-peroxidase method as previously described [34]. Briefly, the tissue was permeabilized using 0.2% Triton X-100, washed in a Tris Buffered Saline (TBS) solution containing 0.5% BSA and were then immersed for 18–20 h in anti-c-Fos oncoprotein polyclonal antiserum specific for c-Fos protein (1∶20.000; Ab-5, Oncogene Sciences, Cambridge MA). Sections were then washed and incubated in biotinylated secondary antibody to rabbit IgG in goat (1∶2000; Vector Laboratories, Peterborough, UK), were washed again and then put into avidin-biotin complex (1∶70; Vectastain Elite ABC Kit, Vector Laboratories). Visualization was by incubation in 2% diaminobenzidine (DAB) solution (Kirkegaard and Perry Laboratories, Gaithersburg, MD). Brain sections were later mounted, dehydrated and coverslipped with Permount (Fisher). Both sides of the bilateral structure were counted and averaged. Digital photographs of coronal sections were taken using the ACT1 program, at ×40 magnification. CFLI cells were identified by dark black immunoreactivity in the nucleus and were counted by an observer blind to the experimental treatment, using the public domain NIH Image-J software. Cell counting was performed on the ARC and DMH (both analyzed from 2 sections containing the largest extent of the nucleus) and the caudal NTS in the hindbrain.

#### Western Blot

ARC punches were extracted from frozen brains, samples were boiled with sample buffer after protein extraction, and total protein was fractionated by 7.5% SDS-PAGE and transferred to nitrocellulose membranes at 4°C. After blocking with 5% skim milk for 3 hours at room temperature, the membranes were incubated with Leptin Receptor antibody (ab5593) or pSTAT3 antibody (Cell signaling) at 4°C overnight (1∶2000 and 1∶200 respectively). The first antibody detects both the short (Ob-Ra) and long form (Ob-Rb) of the leptin receptor. The expression of GAPDH was determined as a loading control. After further incubation with horseradish peroxidase-conjugated secondary (Goat anti-rabbit IgG, SC-2004, Santa Cruz), ECL (Ornat) was added and the signal was detected by gel documentation equipment. The signal intensity was determined by the public domain NIH Image J program. Ventromedial hypothalamus (VMH) punches were also extracted and prepared in a similar manner for the relative quantification of the estrogen receptor alpha (ERα) protein. Similarly, proteins were extracted, and total protein was fractionated by 7.5% SDS-PAGE and transferred to nitrocellulose membranes at 4°C. Blocking was shorter (1 hour at room temperature) with 5% skim milk and then membranes were incubated with ERα antibody (Santa Cruz) at 4°C overnight (1∶1000). The rest was performed exactly as in the leptin receptor protocol.

### Statistical approach

Group differences in DEXA and external measures were analyzed by one way ANOVA comparing the LETO, OdLp (OLETF dam LETO pup), OLETF and LdOp (LETO dam OLETF pups) groups (with post-hoc Duncan's test). For the hormones leptin and insulin, strain differences were pronounced so data were analyzed separately within each pup-genotype (LETO/OLETF). In the leptin and the high fat ENSURE challenges, a similar approach was adopted, examining only the effects of adoption within strains (one-way ANOVA with Duncan's post-hoc tests). Differences in intake between the first and following trials in the high fat challenge within each group were analyzed by paired t-tests. Western blot results were analyzed by one sample t-tests. Estrous cycle structure was analyzed by chi-square test.

## Results

### Fostering reduced long term body weight and food intake in LdOp offspring

**Figure 1:**
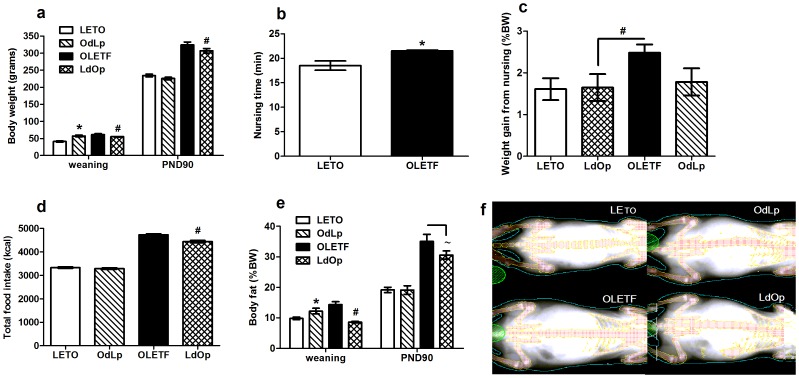
Developmental data. Body weight in grams at weaning (PND22) and PND90 (**A**); nursing time in minutes (**B**); weight gain (%) from nursing (**C**), total food intake in kcal from weaning until PND90 (**D**) and body fat in %BW weaning (PND22) and PND90 (**E**). Representative DEXA pictures of females offspring (**F**). Data are presented in means and SEM. Duncan tests: *p<0.05 for significant differences from LETO controls; # p<0.05 for significant differences from OLETF controls. ∼ for statistical tendency (p = 0.09). N = 6–8 per group.

Body weight (BW) and intake were assessed in females of all groups from weaning and on. Repeated measures analysis of variance (ANOVA) revealed a significant effect of cross-fostering on BW (F(54,24) = 1.88, p<0.05). Overall, fostering did not affect long term BW and food intake in OdLp females, with the exception of BW at weaning (Fig. 1a). LdOp females weighed significantly less in 7 of the 19 days examined, including at weaning and PND90 and consumed less food compared to OLETF females (F(3,24) = 325.59, p<0.001; Duncan's test, p<0.05) (Figs. 1a&d).

### Dams of both strains nursed their litters according to their own genetic background and were not affected by the adopted offspring

On PND18, a nursing test was performed in order to determine the effectiveness of the manipulation, designed to prevent the maternal adaptation reported when whole litters are fostered [28]. Indeed, nursing time differed between the dams, with OLETF nursing longer than LETO dams (Fig. 1b). Overall weight gain (normalized to BW) from nursing bouts tended to differ among the groups. In the LETO offspring, weight gain was similar regardless of the strain of the mother, while LdOp offspring showed decreased weight gain compared to OLETF offspring (Fig. 1c).

### Body composition in the offspring was determined by the early environment at the time of weaning and by genotype at adulthood

Dual energy X-ray absorptiometry (DEXA) was used for determination of body composition and bone morphology. We examined lean and fat mass, bone mineral density, content and area both at weaning (PND22) and PND90. At weaning (F(3,15) = 16.02, p<0.001) and adulthood (F(3,26) = 30.26, p<0.001) percentage body fat differed significantly between the groups. The females' adiposity at weaning was determined by the strain of the mother, but it returned to be genotypically determined at adulthood (Figs. 1d&f). Lean body mass also differed between the groups at the time of weaning and was determined by the strain of the mother (F(3,15) = 6.86, p<0.01) (Table 1a). At adulthood the effect was also significant (F(3,26) = 6.89, p<0.001) and determined by genotype. Bone mineral density (BMD) differed between the groups at weaning (F(3,15) = 14.48, p<0.001) and tended to be significantly different on PND90 (F(3,26) = 2.36, p = 0.094)(Table 1b). A similar profile was observed regarding bone mineral content (BMC); it differed between the groups at weaning (F(3,15) = 15.75, p<0.001), but this difference was not evident at adulthood; OLETF females presented overall lower mineral content than LETO females (Table 1b). Accordingly, bone area differed between the groups at weaning (F(3,15) = 11.17, p<0.001), but not at adulthood (Table 1).

**Table 1:** DEXA parameters at weaning (A) and on PND 90 (B).

(A)				
PND22	LETO	OdLp	OLETF	LdOp
**Lean body mass (gr)**	30.85±1.49	37.07±1.51 *	39.55±1.86 *	37.32±0.65 *
**Bone mineral content (gr)**	0.35±0.02	0.45±0.03 *	0.52±0.02 *	0.54±0.02 *
**Bone area (cm^2^)**	8.365±0.23	9.48±0.43 *	9.87±0.34 *	10.48±0.18 *
**Bone mineral density (cm^2^/gr)**	0.042±0.00	0.048±0.00 *	0.052±0.00 *	0.052±0.00 *
**body length (cm)**	11.15±0.35	13.43±0.30 *	13.08±0.30 *	13.02±0.12

(B)				
PND90	LETO	OdLp	OLETF	LdOp
**Lean body mass (gr)**	101.58±2.53	96.60±2.81	112.88±4.11 *	114.83±3.53 *
**Bone mineral content (gr)**	2.26±0.20	2.00±0.25	1.79±0.28 *	1.58±0.14 *
**Bone area (cm^2^)**	12.38±0.85	10.98±0.91	11.34±1.26	10.41±0.77
**Bone mineral density (cm^2^/gr)**	0.191±0.02	0.183±0.02	0.155±0.01 *	0.152±0.01 *
**body length (cm)**	21.38±0.21	21.00±0.25	22.77±0.14 *	22.13±0.23 *
**insulin (ng/ml**	2.39±0.31	1.47±0.17 *	4.45±1.08 *	4.21±0.70 *
**TNFα (pg/ml)**	547.4±68.6	951.4±146.5 *	945±227.0	777.3±206.6
**IL 1β (ng/ml)**	6.21±0.43	7.57±1.11	6.26±0.58	7.70±0.89
**IL 6 (ng/ml)**	5.12±0.43	2.80±0.73 *	4.70±0.98	5.21±0.89

* p<0.05 for significant differences compared to LETO controls.

### OLETF dams induced a transitory postnatal leptin surge in the LETO offspring, while LETO dams blunted the expected postnatal leptin surge in OLETF offspring

**Figure 2:**
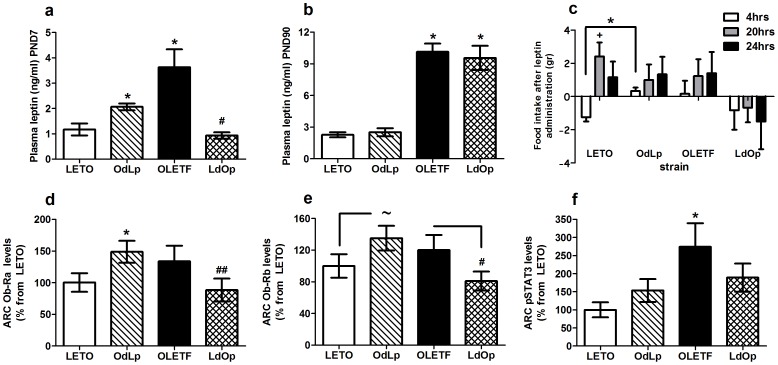
Leptin and leptin receptors. Plasma leptin levels on PND7 (**A**) and on PND90 (**B**); peripheral leptin sensitivity challenge (intake changes in grams, 4, 20 & 24 hours after peripheral leptin injection (2 mg/kg) (normalized to intake after saline injection)) (**C**); protein levels for the long form (Ob-Rb) (**D**) and the short form (Ob-Ra) of the leptin receptors (**E**) and pSTAT3 (**F**) in the ARC. Data are presented in means and SEM. *p<0.05 for significant differences from LETO controls; # p<0.05 and ## p<0.01 for significant differences from OLETF controls; + p<0.05 for significant changes in intake within each group; ∼ = 0.053. N = 5–8 per group.

Previous studies showed that PND7 reflects a critical time window where abnormal leptin levels can lead to life-long metabolic complications [6], [7], [8]. We therefore examined leptin at PND7 and adulthood. In addition, we examined adult insulin levels and further inflammatory markers to assess possible inflammation in lean and obese females with and without childhood hyperadiposity. Plasma leptin levels on PND7 were significantly affected by adoption in both strains (F(3,22) = 12.64, p<0.001), with OdLp females presenting twice as much leptin compared to LETO and with LdOp females presenting normalized (to LETO levels) levels (Fig. 2a). On PND90, leptin was no longer affected by adoption and was low in LETO and high in the OLETF strain (Fig. 2b). While OLETF females showed no alterations in plasma insulin, OdLp females showed significantly lower insulin levels compared to LETO (F(1,9) = 14.26, p<0.01; Table 1b). Overall, TNFα, IL1β & IL6 did not differ between the strains. However, within the LETO strain, higher levels of TNFα (F(1,10) = 6.24, p<0.05) and lower levels of IL6 (F(1,11) = 9.03, p<0.05) were detected in OdLp vs. LETO (Table 1b).

### Adult food intake levels after exogenous leptin administration were determined by the early postnatal environment

We performed a leptin sensitivity test to assess the effects of acute increase in peripheral leptin on food intake in adult females reared in each experimental condition. LETO females presented a normal response (decreased food intake in the first hours following leptin administration) to acute leptin administration, while OdLp females failed to decrease food intake during the first 4 hours after the injection (F(1,9) = 20.90, p<0.001). OLETF females presented complete resistance to leptin, and while LdOp females showed an evident response, the high variability prevented statistical significance (Fig. 2c).

### Ob-Ra and Ob-Rb levels in the Arcuate nucleus (ARC) of the hypothalamus were affected by the early postnatal environment

Next, we examined protein levels of the short (Ob-Ra) and long (Ob-Rb) forms of leptin receptor in adult females of all groups. Additionally, we examined the levels of the phosphorylated transcription factor signal transducer and activator of transcription (pSTAT3), which becomes activated by leptin, to assess Ob-Rb functioning. In the Arcuate nucleus of the hypothalamus (ARC) we found significant differences between the groups in the expression of the short form of Leptin receptor (Ob-Ra). One sample t-test analysis revealed significantly higher Ob-Ra levels in OLETF and OdLp females (t(5) = 2.62, p<0.05 and t(7) = 3.26, p<0.05, respectively) and normalized (to LETO) levels in LdOp females (Fig. 2d). In addition, OdLp females tended to show increased ARC levels of Ob-Rb (t(7) = 2.33, p = 0.052) (Fig. 2e). ARC pSTAT3 levels were significantly higher in OLETF compared to LETO while LdOp and LETO levels did not differ (F(3,24) = 3.22, p<0.05). OdLp pSTAT3 levels were normal (Fig. 2f).

### The sensitivity to leptin in the ARC was determined by the early postnatal environment

**Figure 3:**
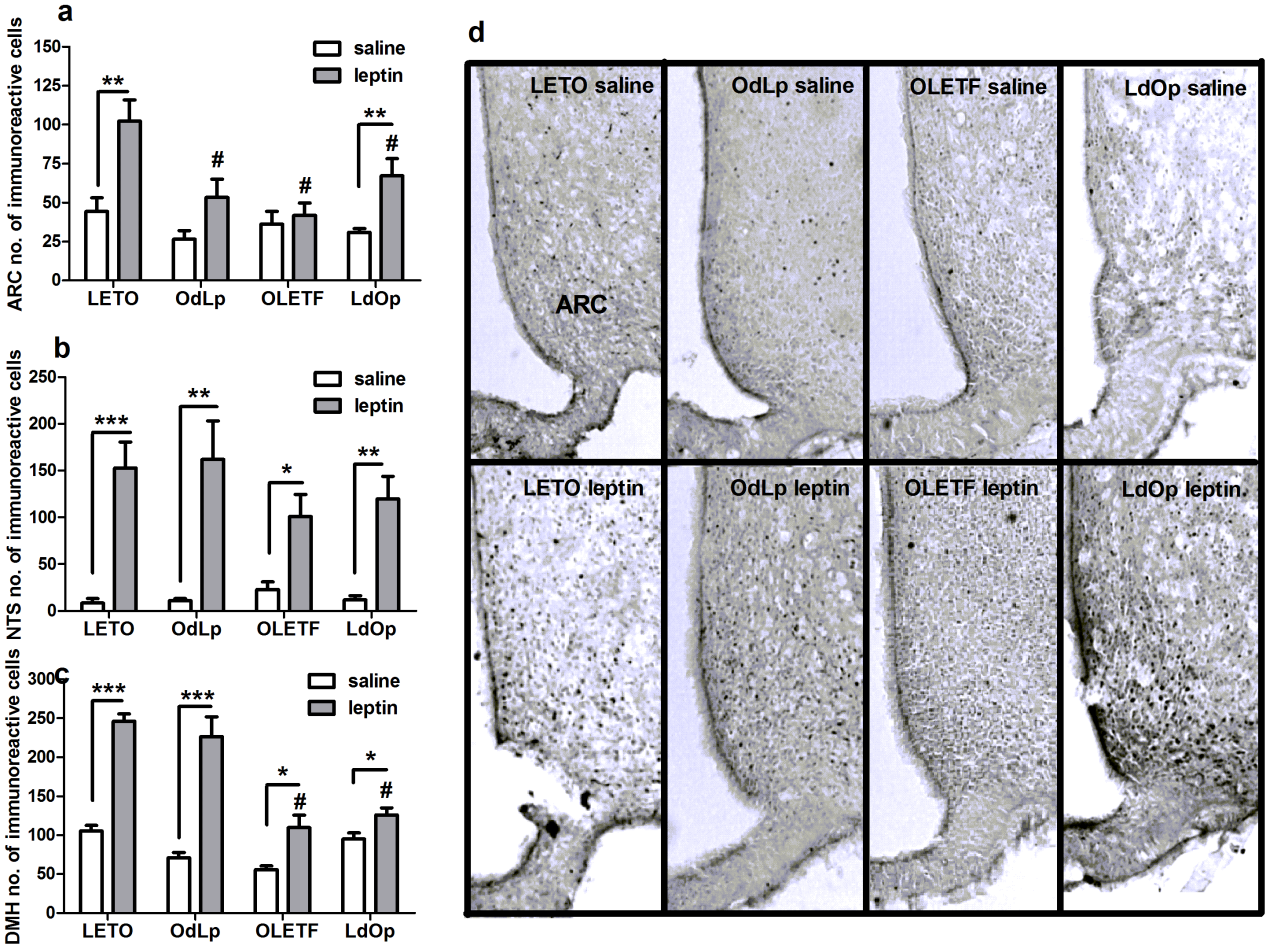
C-fos. C-fos-like immunoreactivity in the ARC (**A**), NTS (**B**) and DMH (**C**) after peripheral saline and leptin administration (2 mg/kg); and representative pictures of the ARC (**D**). *p<0.05, **p<0.01, ***p<0.001, **#**p<0.05 for significant differences from LETO leptin. Data are presented in means and SEM. N = 5–6 per group.

**Figure 4:**
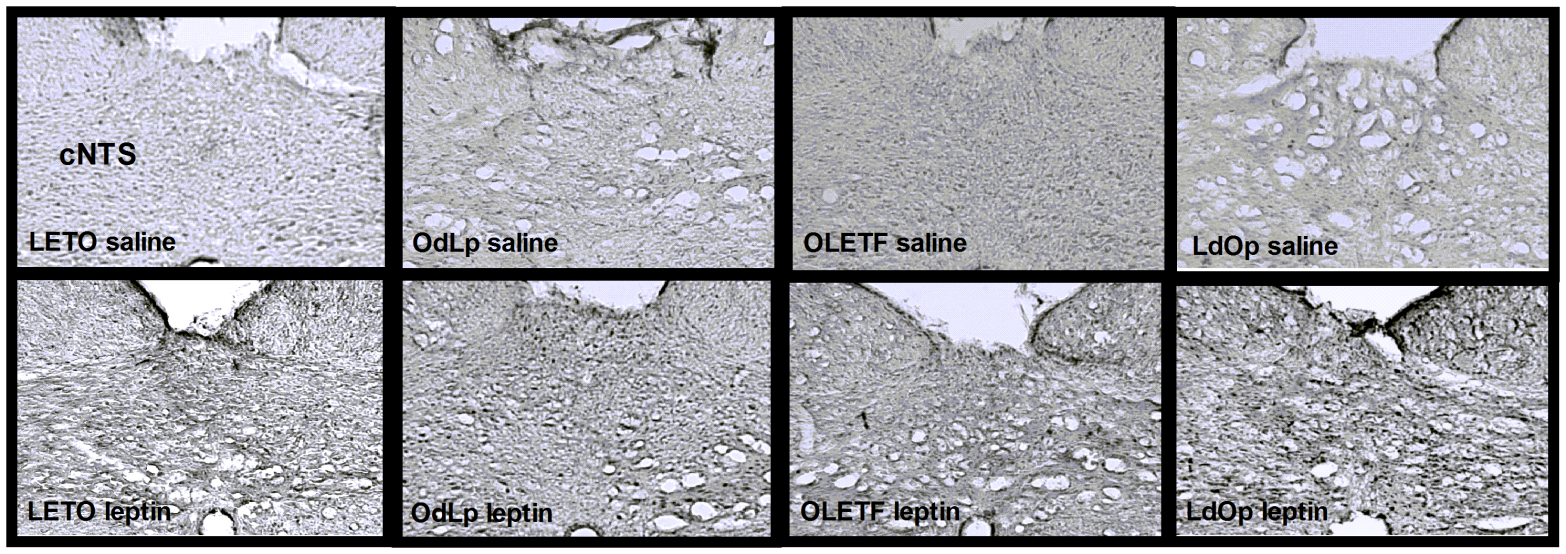
C-fos in the NTS. Representative pictures of the NTS.

**Figure 5:**
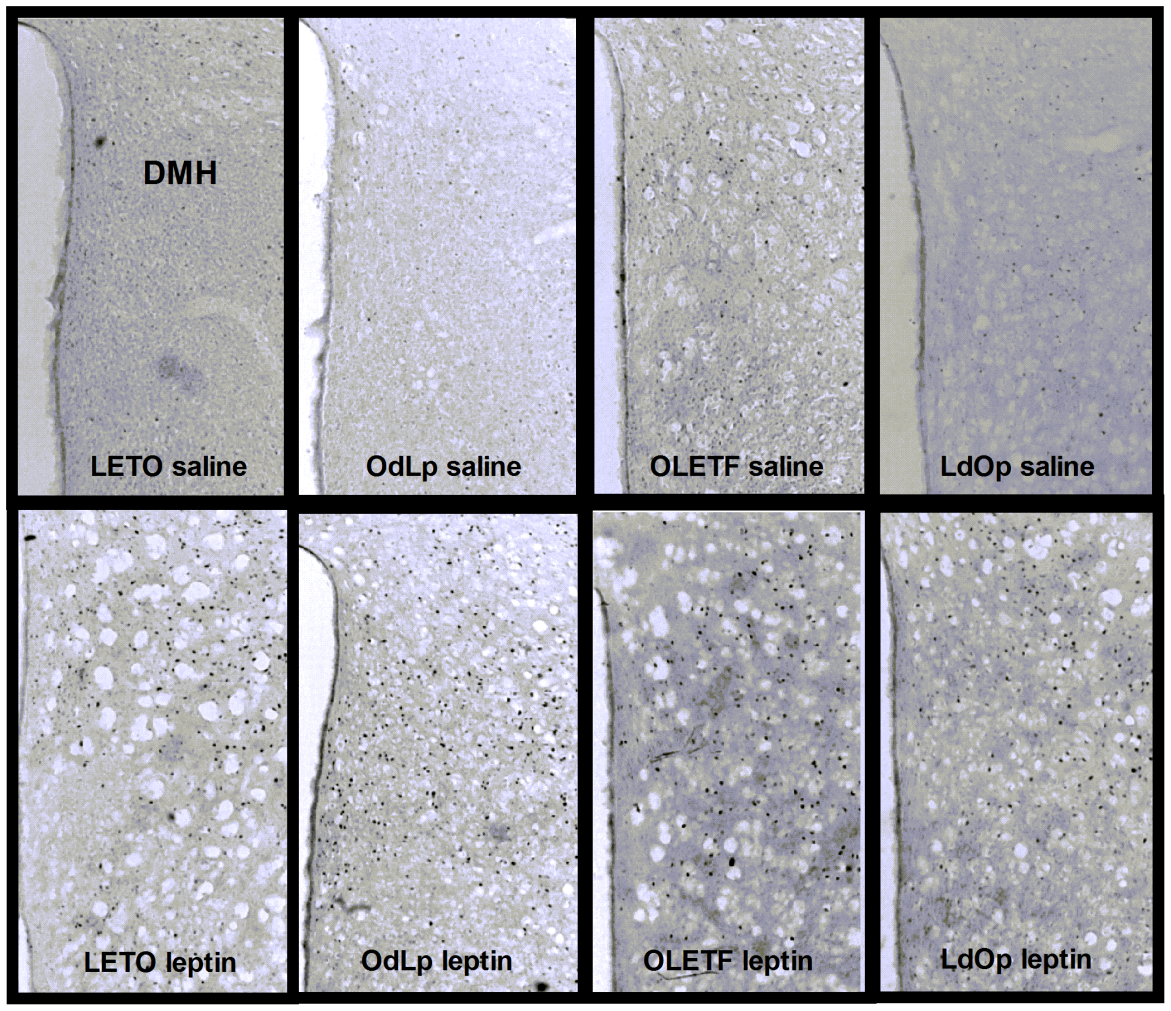
C-fos in the DMH. Representative pictures of the DMH.

To determine the hypothalamic and the brainstem sensitivity to leptin between the groups, we examined c-fos activation in the ARC, in the dorsomedial hypothalamus (DMH) and the nucleus of the solitary tract (NTS) after exogenous leptin administration. The ARC response to leptin was significantly higher in LETO females compared to all other groups (F(3,22) = 6.51, p<0.003, specific between-group differences based on Duncan's test). When compared to same group saline response, activation in response to leptin was significant in the LETO and LdOp groups, while in the OLETF and OdLp groups leptin did not significantly increase c-fos activation (Fig. 3a). In the NTS, leptin significantly and similarly increased c-fos response, compared to saline, in all groups (Fig. 3b). In the DMH, leptin also significantly increased the response in all groups (all t-tests p<0.05) (Fig. 3c). However, c-fos response was much higher in LETO & OdLp (F(3,18) = 16.83, p<0.001) compared to the OLETF strain (Fig. 3c). Representative pictures of the c-fos response in the ARC are shown in fig. 3d. NTS and DMH pictures are shown in figs. 4 & 5.

### OdLp females presented hyperphagia when challenged with a high fat/palatable liquid, while LdOp females moderated their intake across the challenge trials

**Figure 6:**
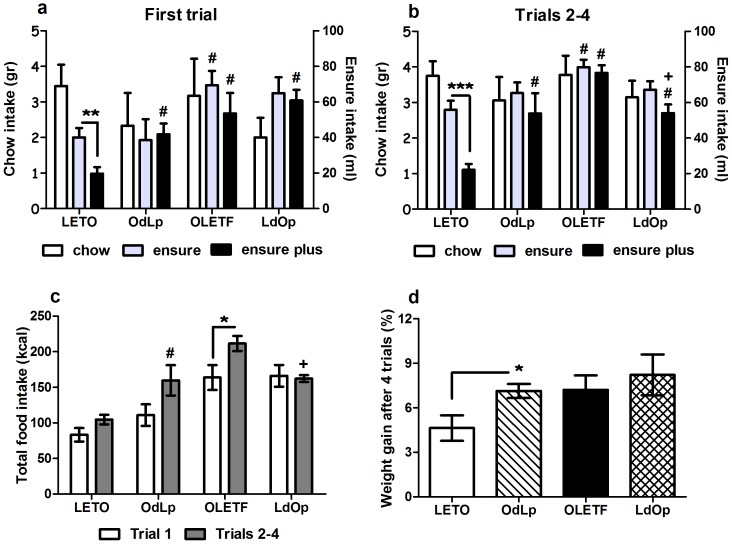
ENSURE high fat challenge. Intake of standard chow in grams (left y-axis), ENSURE and ENSURE plus in ml (right y-axis) after a 12 hour overnight trial. Intake after the first trial (**A**); intake after trials 2–4 (**B**); total caloric intake (**C**) and weight gain after the 4 trials (in percentages) (**D**). Data are presented in means and SEM. Duncan tests: *p<0.05,**p<0.01, ***p<0.001. #p<0.05 for significant differences from LETO controls and +p<0.05 for significant differences from OLETF controls within each food type. N = 6–9 per group.

The observed alterations in leptin sensitivity were tested in a food challenge, where females were provided with the option to consume unlimited amounts of two palatable liquids: ENSURE vanilla (20% fat), ENSURE PLUS vanilla (30% fat) and standard chow, overnight, for four nights. In the first trial, intake of the unfamiliar liquids reflects short term satiety and functioning of the orosensory signals. In the following trials, long term satiety signals, such as leptin, become involved. Both in the first and the following trials, LETO females showed a clear preference for the low-fat ENSURE (based on paired t-tests). OdLp, LdOp and OLETF females showed no preference for any of the liquids in any of the trials (Figs. 6a&b). In addition, one-way ANOVA revealed significant differences between the groups in the amounts of ENSURE (F(3,26) = 3.51, p<0.05 and F(3,26) = 3.48, p<0.05) and ENSURE PLUS (F(3,26) = 8.45, p<0.001 and F(3,26) = 14.47, p<0.001) consumed in the first and following trials (respectively). Specifically, LETO and LdOp females maintained their caloric intake, OLETF females increased their intake across the trials (t(5) = −3.75, p<0.05) and OdLp females showed a strong tendency in the same direction (Figs. 6a&b). Regarding cumulative caloric intake, levels differed among the groups both in the first (F(3,26) = 8.94, p<0.001) and following trials (F(3,26) = 16.72, p<0.001) (Fig. 6c). Finally, body weight gain was significantly higher in OdLp vs. LETO females after the 4-day challenge, reaching OLETF-like levels (F(3,26) = 3.00, p<0.05) (Fig. 6d).

### The estrous cycle structure and pattern of intake were affected by the early environment

**Figure 7:**
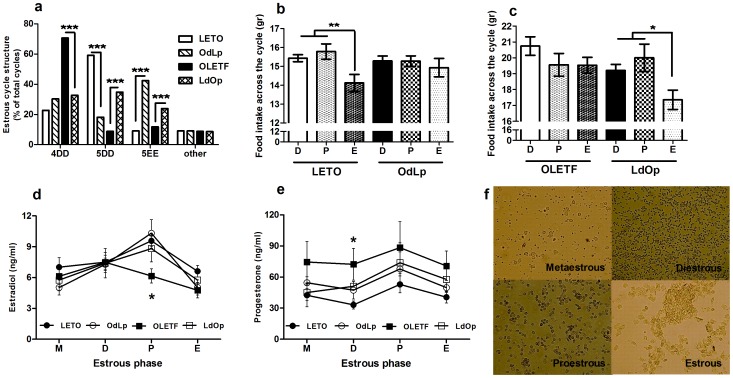
Females' estrous cycle parameters. Structure (data are presented in percentages of total cycles examined) (**A**); Intake (in grams) across the estrous cycle in LETO (**B**) and OLETF (**C**) females; feces estradiol (**D**) and progesterone (**E**) levels across the estrous cycle. Representative pictures of the estrous phases (**F**). *p<0.05, **p<0.01, ***p<0.001. N = 11–12 per group. 4D: 4 day cycles, 5DD: 5day double diestrous cycle and 5EE: 5 day double estrous cycles.

Given the involvement of leptin in reproduction, we examined the structure of the estrous cycle and respective intake patterns. Overall, fostering significantly affected the structure of the cycle. In OdLp rats compared to LETO rats (chi-square = 16.97, df = 2, p<0.001), the amount of 4-day cycles remained unaffected, the 5-day double diestrous cycles were less frequent and 5-day double estrous cycles were increased. In the OLETF strain, changes in the estrous cycle were also evident (chi-square = 14.15, df = 2, p<0.001), with drastically reduced frequency of 4-day cycles in LdOp compared to OLETF females, but significantly increased frequency of 5-day double diestrous and 5-day double estrous cycles (Fig. 7a). The day of full vaginal opening did not differ among the groups. Food intake was also affected by adoption. LETO females showed the expected reduction in food intake in the estrous phase of the cycle, but this reduction was abolished in the OdLp females (Fig. 7b). Similarly, adoption fixed the food intake profile across the cycle in LdOp females compared to OLETF controls, which showed no significant intake reduction in the estrous phase of the cycle (Fig. 7c).

### Fostering normalized the otherwise abnormal levels of estradiol in the proestrous phase of the cycle in OLETF females

The female hormones are known to interact with leptin and have a strong influence on the fluctuations in appetite across the reproductive cycle. Given the alterations in the estrous cycle and intake observed among the groups and, in OLETF, the known fertility problems, we examined the female hormonal profile across the cycle by analyzing estradiol and progesterone metabolites in the feces. The levels of estradiol tended to differ between the groups only during the pro-estrous phase of the cycle (p = 0.052). Specifically, OLETF females presented significantly lower levels than LETO (F(1,16) = 14.75, p<0.001), while LdOp females showed estradiol levels similar to LETO controls (Fig. 7e). Progesterone levels differed between the groups only in the diestrous phase of the cycle (F(3,18) = 3.97, p<0.05). OLETF females presented higher progesterone levels than LETO during the diestrous phase (F(1,8) = 9.00, p<0.05) and tended to have higher levels both in the pro-estrous phase (p = 0.065) and estrous (p = 0.061) phases. Both fostering groups fell in-between, showing no significant differences from any of the control groups (Fig. 7f).

## Discussion

### Alterations in leptin functioning as a result of postnatal hyperleptinemia and hyperadiposity

The food preference test revealed that wild type females can be strongly affected by an early obese environment leading to an acquired predisposition to diet induced obesity in the presence of palatable food. While LETO control females clearly preferred the low-fat ENSURE, OdLp females' over-consumption of the high fat liquid, which was gradually exacerbated across the trials, induced significant weight gain exposing a potential predisposition to diet induced obesity. OLETF rats failed to discriminate between the high fat and the low fat liquids regardless of the early postnatal environment, but LdOp females managed to limit overall food intake during the progressive food challenge trials, implying a relative moderation in their hyperphagia even in the presence of the strong genetic tendency to obesity.

The present study further demonstrates that an early obesogenic postnatal environment can induce selective peripheral leptin resistance in the ARC, without affecting leptin signaling proceeding from the vagal pathway. Both the nucleus of the solitary tract (NTS) (and the nodose ganglia on the vagal nerve) and the ARC express both the long and short forms of leptin receptors [35], suggesting two pathways of action of leptin in the brain. Sensory signals from the gastrointestinal tract (GI) and associated digestive viscera are delivered to the brain primarily by vagal afferents that terminate centrally within the caudal NTS (cNTS) [36]; and previous studies reported dense axonal projections from the cNTS to the dorsomedial hypothalamus [37]. The ARC on the other hand, responds primarily to leptin signaling originating in adipose tissue. Leptin receptors in the ARC responding to circulating leptin represent the main pathway and leptin receptors on vagal afferent fibers (a portion of which exert its actions through interactions with CCK1R [38]) transmitting information from the GI are a further, secondary pathway.

Resistance to leptin can develop by at least two different mechanisms [39], either circulating leptin fails to reach its targets in the brain (peripheral leptin resistance) [40] [41] or when there is a failure of components of the intracellular Ob-Rb signaling cascade (hypothalamic leptin resistance) [8] [42]. There is a lack of consensus regarding the actual cause of peripheral leptin resistance, which might result either from a reduction in the expression of Ob-Rb (the long form of leptin receptor, responsible for all the biological actions) [7], reduction in Ob-Ra (the short form of leptin receptor, believed to be responsible for leptin transport into the brain) [43], [44] or alternatively, from the saturation/malfunctioning of the existing receptors [40], [45], [46]. In some cases, malfunctioning of Ob-Rb in adult offspring resulting from early postnatal leptin administration [46] or high fat diet during pregnancy and lactation [45] was accompanied by up-regulation in the expression of leptin receptors. One study performed on diet induced obese mice suggested that leptin resistance resulted from selective insensitivity of the ARC to circulating levels of leptin, while other brain areas remained responsive [12]. Ob-Rb presents two tyrosine residues that become phosphorylated during receptor activation, which mediate distinct signaling pathways as follows: SHP-2 binding to Tyr985 positively regulates the ERK/MAPK/c-fos pathway and STAT3 binding to Tyr1138 mediates the inhibitory SOCS3 pathway [47]. We examined pSTAT3 levels and c-fos activation in the ARC in order to examine both pathways as possible contributors to the alterations in leptin sensitivity.

Regarding c-fos in the vagal pathway, the DMH and NTS response to leptin remained intact in LETO females regardless of the adoption condition. In contrast, the ARC c-fos response was determined by the postnatal environment. Accordingly, OdLp females showed reduced ARC c-fos response to leptin that correlated with the blunted response to peripheral leptin in the behavioral sensitivity test. Ob-R receptors were highly expressed in the ARC of these females probably as a consequence of the early leptin surge (similarly to control OLETF females). In the OLETF strain, c-fos response in the NTS was relatively low but still within the normal range. In the DMH, the only hypothalamic area naturally expressing CCK_1_ receptors in wild type rats [48], c-fos activation was decreased regardless of adoption, implying the need for functional CCK_1_ receptors in order to adequately activate this brain area. In the ARC, the c-fos response to leptin was sharply improved in LdOp females, despite their hyperleptinemia at the time of sacrifice; and ARC Ob-Rb were normalized to LETO levels. To our knowledge, only one study examined central leptin sensitivity in OLETF (male) rats. Their conclusions suggested that the OLETFs' central leptin sensitivity was intact despite evident peripheral insensitivity and hyperleptinemia. However, the study lacked any statistical findings and the pictures presented reflected impaired central leptin sensitivity, contrary to their conclusions [24]. In another study, Ob-Rb levels were reportedly reduced in OLETF males subjected to food restriction, but leptin sensitivity was not directly examined [49]. LdOp females, despite being obese and hyperleptinemic as adults, showed an improved response resulting from re-gained sensitivity of ARC neurons to circulating leptin. Overall, high levels of Ob-Rb protein were accompanied by high levels of pSTAT3 (suggesting normal activation) so the option of early receptor saturation was discarded. Given that c-fos response in the ARC of OLETF and OdLp females was abnormal, we conclude that the ERK/MAPK/c-fos pathway was the one susceptible to early hyperleptinemia. The specific location of the alteration in this particular cascade will be examined in future studies.

### Implications of postnatal hyperleptinemia/obesity to female health

OLETF females present an abnormal estrous cycle structure [25], [26], abnormal intake across the cycle [26], [28] and disrupted hormonal profile and fertility complications [25] which are consistent with pathologies often found in obese women. Estrous cycle structure, hormonal profile and food intake are also abnormal in obese Koletsky rats, which have leptin receptor gene mutations. Specifically, these rats show high progesterone and low estradiol levels and hyperphagia, effects that can be fixed by insertion of functional leptin receptors by viral therapy specifically in the ARC [50]. Leptin signaling in the ARC is also critical for restoring estrous cyclicity as shown in a study using viral insertion in Zucker rats [51].

In our study, we examined the long term effects of the early postnatal environment on most of the mentioned parameters. At adulthood, OdLp females were characterized by normal adiposity/body weight/food intake profile, but showed a permanent disruption in the estrous cycle structure and intake profile across the cycle. Given normal estradiol and estradiol receptor alpha levels in the VMH of these females (not shown), we suggest that their abnormal food intake pattern across the cycle may be related to impaired leptin sensitivity. One well-accepted role of leptin is to act as a gatekeeper to a range of activities that are not essential for immediate survival, such as reproduction [25]. The normalization of the estrous cycle structure to the strain of the adopting mother also has potential implications for the fertility problems of the OLETF females. One previous study has related their fertility complications to the hyperleptinemia these females present as adults. Moreover, their abnormal estrous cycle structure was accompanied by abnormal LH/prolactin surges in response to estradiol [25]. We suggest that fertility and hormonal related issues may be determined much earlier in life than previously believed. In addition, normalization of intake across the cycle in LdOp females may results from the significant improvement in their hormonal profile (especially the estradiol surge in the proestrous phase) together with improved sensitivity to leptin. Our results may complement those reported by Watanobe et al [25]: The lack of LH/prolactin response to E_2_ administration in hyperleptinemic animals may also be related to early-acquired leptin resistance and the low endogenous estradiol secretions during proestrous in OLETF females. In addition, the anorectic effects of estradiol during the estrous phase of the estrous cycle are believed to rely on an increase in the central sensitivity to CCK and leptin [52]. It is very surprising then, that intake across the cycle in these females appeared to be mostly determined by developmental parameters rather than the actual levels of leptin or an intact CCK system at adulthood. This highlights the need for intact leptin sensitivity as a key feature required for the correct behavioral response to hormonal changes across the estrous cycle. While no fertility assessments were performed in the present study, our findings suggest a new concept regarding leptin involvement in reproduction and highlight the importance of the early nutritional status by linking fertility-related female health to normal functioning of the leptin system rather than to obesity (and the accompanying hyperleptinemia) itself.

One further aspect regarding the females' health was the issue of bone morphology. Visceral fat is increasingly recognized as a determinant (inversely correlated) of bone mineral density (BMD) [53], [54], an association that may be mediated by adipokines, such as adiponectin and leptin, and inflammatory fat products such as IGF-1, IL-6, IL-1β and TNFα. Chronic inflammation is deleterious to bone and may be related to a predisposition to osteoporosis in obese women [53]. In this context, we examined BMD (and mineral content and bone area) at weaning and adulthood, as well as circulating levels of IL-6, IL-1β and TNFα. OLETF females presented increased BMD, BMC and bone area at weaning and reduced BMD and BMC levels at PND90 compared to LETO controls. Given that LdOp females showed no improvement in these parameters in the long term we concluded that the pre-requisites for osteoporosis result from chronic obesity rather than obesity & leptin levels during the early postnatal period. Additionally, IL-6, IL-1β and TNFα did not differ significantly between OLETF and LETO controls on PND90, but OdLp females showed higher TNFα and lower IL-6, despite normal levels of leptin and adiposity and intact BMD. We can only hypothesize about the implications of these alterations, which may predispose these females to increased inflammation given an immunological challenge. OLETF females only tended to higher TNFα levels but showed normal IL-6 and IL-1β despite being chronically obese and with hypertrophic adipocytes [15], [26]. This profile is similar to the one found in OLETF males, who also showed higher TNFα levels but IL-1β levels similar to LETO controls in a different study [55]. Given the high levels of inflammatory cytokines usually found in obesity, it is likely that their levels will probably rise as females' approach the age of 4 months, an age when they usually become morbidly obese [26].

In sum, adult OdLp females were characterized by a normal phenotype, but showed a permanent disruption in estrous cycle parameters, decreased response to leptin and sensitivity to diet induced obesity when exposed to an unbalanced/palatable diet. LdOp females presented reduced BW/intake/body fat at adulthood, normal estrous parameters, improved peripheral leptin sensitivity and remarkable self-moderation when given the chance to overconsume ENSURE. Altogether, it appears that the emergence of morbid obesity in the OLETF strain is either caused or accompanied by the disruption of the leptin system, which worsens their innate hyperphagic phenotype. More importantly, the pattern found in the OdLp offspring highlights the importance of the lactating period on female development, showing that manipulations at this time window can potentially disrupt the development of an intact leptin/reproductive system and apparently induce sensitivity to diet induced obesity, when challenged, even in the absence of a supporting genetic predisposition.
